# An oral health intervention for people with serious mental illness (Three Shires Early Intervention Dental Trial): study protocol for a randomised controlled trial

**DOI:** 10.1186/1745-6215-14-158

**Published:** 2013-05-29

**Authors:** Hannah F Jones, Clive E Adams, Andrew Clifton, Jayne Simpson, Graeme Tosh, Peter F Liddle, Patrick Callaghan, Min Yang, Boliang Guo, Vivek Furtado

**Affiliations:** 1 CLAHRC-NDL, Institute of Mental Health, University of Nottingham, Triumph Road, Nottingham, NG7 2TU, UK; 2 Cochrane Schizophrenia Group, Institute of Mental Health, University of Nottingham, Triumph Road, Nottingham, NG7 2TU, UK; 3 School of Human & Health Sciences, University of Huddersfield, Queensgate, Huddersfield, HD1 3DH, UK; 4 Ferham Clinic, Kimberworth Road, Rotherham, S61 1SAJ, UK; 5 Division of Psychiatry, Institute of Mental Health, University of Nottingham, Triumph Road, Nottingham, NG7 2TU, UK; 6 School of Nursing, Midwifery and Physiotherapy, Queen's Medical Centre, Derby Road, Nottingham, NG7 2UH, UK

## Abstract

**Background:**

Oral health is an important part of general physical health and is essential for self-esteem, self-confidence and overall quality of life. There is a well-established link between mental illness and poor oral health. Oral health problems are not generally well recognized by mental health professionals and many patients experience barriers to treatment.

**Methods/Design:**

This is the protocol for a pragmatic cluster randomised trial that has been designed to fit within standard care. Dental awareness training for care co-ordinators plus a dental checklist for service users in addition to standard care will be compared with standard care alone for people with mental illness. The checklist consists of questions about service users’ current oral health routine and condition. Ten Early Intervention in Psychosis (EIP) teams in Nottinghamshire, Derbyshire and Lincolnshire will be cluster randomised (five to intervention and five to standard care) in blocks accounting for location and size of caseload. The oral health of the service users will be monitored for one year after randomisation.

**Trial registration:**

Current Controlled Trials ISRCTN63382258.

## Background

Oral health is an important part of physical health and is essential for self-esteem, self-confidence and overall quality of life [1,2]. Oral diseases are the most common chronic disease and have a great impact on individuals [3]. Oral health is not just about having healthy teeth it is a ‘standard of health of the oral and related tissues which enables an individual to eat, speak and socialise without active disease, discomfort or embarrassment and which contributes to general well-being’ [1]. It has often been the case that when a person has a serious mental illness and are unwell, their oral health may not be seen as a priority, so it is neglected and deteriorates [2]. Oral health problems are often not detected by mental health professionals [4] and, to compound the matter, many dentists shy away from treating people with psychosis [5,6].

Research conducted in the last decade that has examined the oral health of people with serious mental illness has generally concluded that oral health for people with serious mental illness is poor compared with the general population [7-11]. People with serious mental illness are likely to experience more oral health problems and require more dental treatment than people from the general population [2]. Side effects of medication prescribed to people with serious mental illness can cause a lack of saliva, which in itself can lead to caries [12-15].

### Guidelines

The British Society for Disability and Oral Health guidelines published in 2000 made a number of recommendations for oral health care for people with mental health problems, including providing oral health advice, support, promotion and education addressing the oral health needs of clients [2]. We are not aware of other guidelines regarding dental care for people who have been diagnosed with serious mental illness, practical advice is missing.

### Surveys

Recent surveys that have described the current oral health of people with serious mental illness in various settings, have concluded that the oral health of people with serious mental illness is poor, and many have indicated that it is significantly worse than the general population [16].

### Systematic reviews

A recent systematic review and meta-analysis of advanced dental disease in people with severe mental illness found that people with mental illness were 3.4 times more likely to have lost teeth than the general population and had higher rates of decayed, missing or filled teeth [17]. A meta-analysis of 57 studies looking at the prevalence of suboptimal oral health of people with mental illness and describing approaches to promote oral health [18], found a suboptimal oral health prevalence of 61% as well as highlighting the need for oral health training for mental health professionals. A recent Cochrane review [19] found no relevant randomised trials comparing an oral health advice intervention with standard care for people with serious mental illness that met the inclusion criteria. Another Cochrane review assessed the effectiveness of physical health monitoring as a means of reducing morbidity, mortality and improving quality of life in people with serious mental illness but also found no trials meeting the inclusion criteria [20].

### Trials

There are very few randomised controlled trials of monitoring or advice regarding physical health care for people with serious mental illness. One small trial randomised sixty people to interventions of motivational interviewing plus oral health education or oral health education alone and found that the oral health of individuals who received the motivational interviewing improved significantly more compared to oral health education alone [14]. Another trial randomly assigned 100 participants to receive professional hygienic training or to a no treatment control group and also compared the effectiveness of the intervention for those treated with classical neuroleptics and atypical neuroleptics [21]. Results showed that the effectiveness of oral hygiene training for people receiving classical neuroleptics was lower than for those receiving atypical neuroleptics.

### Objectives

To examine whether dental awareness training plus a dental checklist leads to a clinically significant difference in oral health behaviour of people with serious mental illness.

### Design

#### Setting

The trial will be conducted as part of standard care provided by the Early Intervention in Psychosis (EIP) teams in Nottinghamshire, Derbyshire and Lincolnshire (UK). These three shires are located in the East Midlands of England and each have a mixture of urban and rural areas with a diverse population. The EIP teams provide intensive treatment and support to people with a first experience of symptoms such as hearing voices or those who develop unusual beliefs which may indicate the onset of psychosis. Care co-ordinators are the main contact person for service users throughout their involvement with the service; it is the care co-ordinators who will be delivering the intervention in this trial to their service users.

The three shires dental trial was designed with considerable collaboration with EIP clinicians and service users to make it acceptable to be delivered with minimal disruption alongside standard care. The dental checklist (see Additional file 1) was adapted from the British Society for Disability and Oral Health (BSDH) guidelines [2] in design workshops with researchers, clinicians, service users and carers.

#### Sample size

No previous trials exist [19]. It is, therefore, difficult to determine the number of people that need to be recruited to generate clinically significant data regarding the effect of an oral health advice intervention on the oral health of people with a serious mental illness. As we designed the study in consultation with clinicians and service users, we tried to gain an impression of the size of difference that would cause a change in practice. We did not formally record these estimates, but consensus suggested a range of between 10% to 20%. We estimate the sample size for the mid-way point (Table 1).

**Table 1:** Sample size needed to detect an absolute difference of 15% in the proportion of visits to a dentist (α = 5%, power = 80%)

**Standard care, %**	**Dental intervention, %**	**Non-cluster N (total)**	**Multiplied by design effect**	**Adjusted for 20% dropout**
5	20	176	334	418
10	25	226	430	538
15	30	268	510	638
20	35	302	574	718
25	40	330	628	785
30	45	352	670	837
35	50	366	696	870

Complicating this further is that this is a cluster trial so the EIP teams will be randomised as a whole team rather than at the individual patient level. Intraclass correlation coefficient (ICC) in cluster trials are difficult to estimate if no previous studies exist and to not take clustering into account would lead to ignoring the potential for a unit-of-analysis error [22-24]. Simply estimating that we will be able to randomise the people for whom the 10 teams provide care into two groups is not an accurate reflection of the power of the study. This has to be multiplied by a design effect to adjust for clustering. There are two levels of clustering within this trial, the EIP teams are the clusters that will be randomly allocated to receive the interventions but there are also clusters within the teams; the individual care co-ordinators. Non-cluster N was calculated using the Stata version 11 software (StataCorp, College Station, TX, USA) with alpha = 0.05, power = 0.80. The design effect (DE) was calculated considering patients clustered within each staff/team and each staff sees 10 patients (DE = 1+ (10–1)*0.1 = 1.9), with an intracluster coefficient of 0.1 as a best guess.

Assuming that 5% of the service users who receive standard care visit a dentist, to then detect a 15% increase in the proportion of those who visit a dentist in the dental intervention arm with 80% power at 0.05 significance level, we need 176 service users for a single-centre trial, after adjusting for the cluster effect by multiplying this by the design effect, we need to recruit 334 service users. After further adjusting for 20% of service users lost to follow up, the final number of service users we need to recruit is 418. Other situations with various proportions of visits to a dentist in standard care are also presented in Table 1.

All these assumptions we have made in this power calculation may be correct or all may be incorrect. It is, at this point, quite impossible to say. As can be seen from the background, no studies have been undertaken in this area. There is no data to even guess what any effect may or may not be. This is a pioneering trial that will set a standard and will allow researchers in the future to have some benchmark off which to work.

#### Ethical considerations

EIP team managers will be asked to consent to the trial being conducted within their teams. Informed consent will be sought from care co-ordinators in accordance with Research Ethics Committee (REC) and Good Clinical Practice (GCP) guidance. Service users will be asked by the care co-ordinator if they agree to the care co-ordinator completing the dental checklist at the time of their regular appointment. Service users will not give formal consent for this trial, agreeing to answer the questions on the dental checklists is their consent. This is standard for routine care and public health measures. This is due to the intervention being aimed at the care co-ordinators who receive the dental awareness training, and it is the effect of this training and the checklist that is being measured and not the responses of each service user. The effect of this combined intervention will influence the service users’ dental awareness, even if they do not complete the checklist. It will be made clear to the service users that they do not have to answer the questions if they wish and this will have no detrimental effect on their standard care. If the need arises, relevant information only will be disclosed to the care co-ordinator or another relevant individual in authority.

The trial has received a favourable opinion from the East Midlands Nottingham Research Ethics Committee (REC reference 11/EM/0205) and from the Nottinghamshire, Derbyshire and Lincolnshire National Health Service (NHS) Research and Development (R&D) departments. The trial team have research passports, GCP training, Criminal Records Bureau checks and letters of access for each NHS Trust.

All data will be made anonymous and stored securely. Changes to the protocol will not be implemented until the amendments or revised documents have received favourable opinion from the REC and R&D departments. A protocol amendment intended to eliminate an apparent immediate hazard to participants may be implemented immediately and the REC will be notified as soon as possible to request approval. Minor protocol amendments for logistical or administrative reasons may be implemented immediately and the REC will be informed.

## Methods

### Randomisation

This is a pragmatic, open, cluster randomised controlled trial. We are limited by the number and size of the teams as well as by the compliance of the care co-ordinators. The Nottingham Clinical Trials Unit (CTU) will create a randomisation program that will be used by the researcher to randomise the EIP teams. The researcher will list the teams in such a way as to anonymise them, we will then block randomise; the block being the number of teams within each county. This will ensure that each county gets some degree of exposure to the intervention. EIP teams will be grouped into pairs according to location and size of team, which will then be randomised with one team being allocated the active intervention and the other the control. Randomisation will be stratified to ensure that both of the interventions are roughly equal in terms of team location, number of care co-ordinators within the team and size of caseloads. The CTU will keep full records of the procedure, but there will be no requirement for having procedures for breaking code.

### Participants

Care co-ordinators will be recruited by the EIP team managers and the trial team, and service users will be recruited by the care co-ordinators. Care co-ordinators will be informed of all aspects concerning participation in the trial.

#### Inclusion criteria

Any EIP team in Nottinghamshire, Derbyshire and Lincolnshire and any service users under the care of a care-coordinator in one of these teams, who is aged 18 years or above will be included. Any concomitant treatments are allowed.

#### Exclusion criteria

Any EIP team that does not wish to take part and any individual care co-ordinator who feels that they do not wish to take part will be excluded. The data from service users under the age of 18 at randomisation will not be collected. People who are unable to provide informed consent will be excluded.

### Removal of participants from therapy or assessments

Teams or care co-ordinators within each team can withdraw at any time. They will have given consent but could decide to withdraw that consent. All data up to the point of withdrawal will be used. Withdrawal from the study would result in resumption of standard care. Service users will be withdrawn if a team or care co-ordinator removes their consent.

Participants may be withdrawn from the trial either at their own request or at the discretion of the Investigator. Service users will be made aware that this will not affect their future care. All participants will be made aware (via the information sheet and consent forms) that should they withdraw the data collected to date cannot be erased and may still be used in the final analysis.

### Procedure

Before teams are randomised, all managers will be given information sheets and asked to sign a consent form providing permission for their team to be involved in the trial. We will collect demographic information about the EIP teams to create a list of eligible service users and a cross-coding sheet by which each service user can be allocated an anonymous trial ID. Demographic information will include team location, number of care co-ordinators within the team, size of caseloads and distance to dental services from the team base. The trial team will not have access to identifiable NHS data.

#### Dental intervention

After randomisation, EIP teams allocated to receive the dental intervention will be approached by the trial team to arrange a convenient time to conduct the one-off dental awareness training at the start of the 12-month intervention period. Information sheets will be given out to care co-ordinators and consent forms will be signed. This will fit within the usual multidisciplinary team meetings, but is likely to take around 30 minutes, including time for questions and shared experiences. There will be a manual in order to keep it consistent for all teams (see Additional file 2). The training will briefly cover the aims and background of the trial, how to complete the checklist, service user ID number allocation, how to return the completed checklists to the trial team and discussion about what to do in certain situations regarding adverse events. The care co-ordinators will be encouraged to use the checklist for all their service users at their earliest convenience. The checklist is printed on carbonless copy paper, one copy of the dental checklist will be kept in the service users’ notes and one will be returned in pre-paid envelopes to the trial team. Care co-ordinators will also be encouraged to offer their service users an oral hygiene information sheet which contains oral hygiene tips and information on how to find an NHS dentist (see Additional file 3).

#### Control group

EIP teams allocated to the control group will continue to deliver standard care for 12 months. They will receive the dental awareness training and will be asked to use the dental checklist one year after the intervention group, following the same procedure as the intervention group. Care co-ordinators in control teams will be given information sheets and asked to sign consent forms.

#### Follow-up

The trial team will prompt the intervention group care co-ordinators for the 12-month follow-up where dental checklists will be completed again for all service users. A total of 100 service users will be randomly selected from all teams and their care co-ordinators will be encouraged to ask them if contact from the trial team is acceptable regarding the Oral Impacts on Daily Performance (OIDP) measure as well as for collecting detailed data from their dentists (if they have visited a dentist within the previous 12 months). If it is not - no contact will be made. For this person, the trial is then over. The OIDP is a scale which assesses the impacts to which dental problems affect an individual’s life on a daily basis (see Additional file 4).

**Figure 1:**
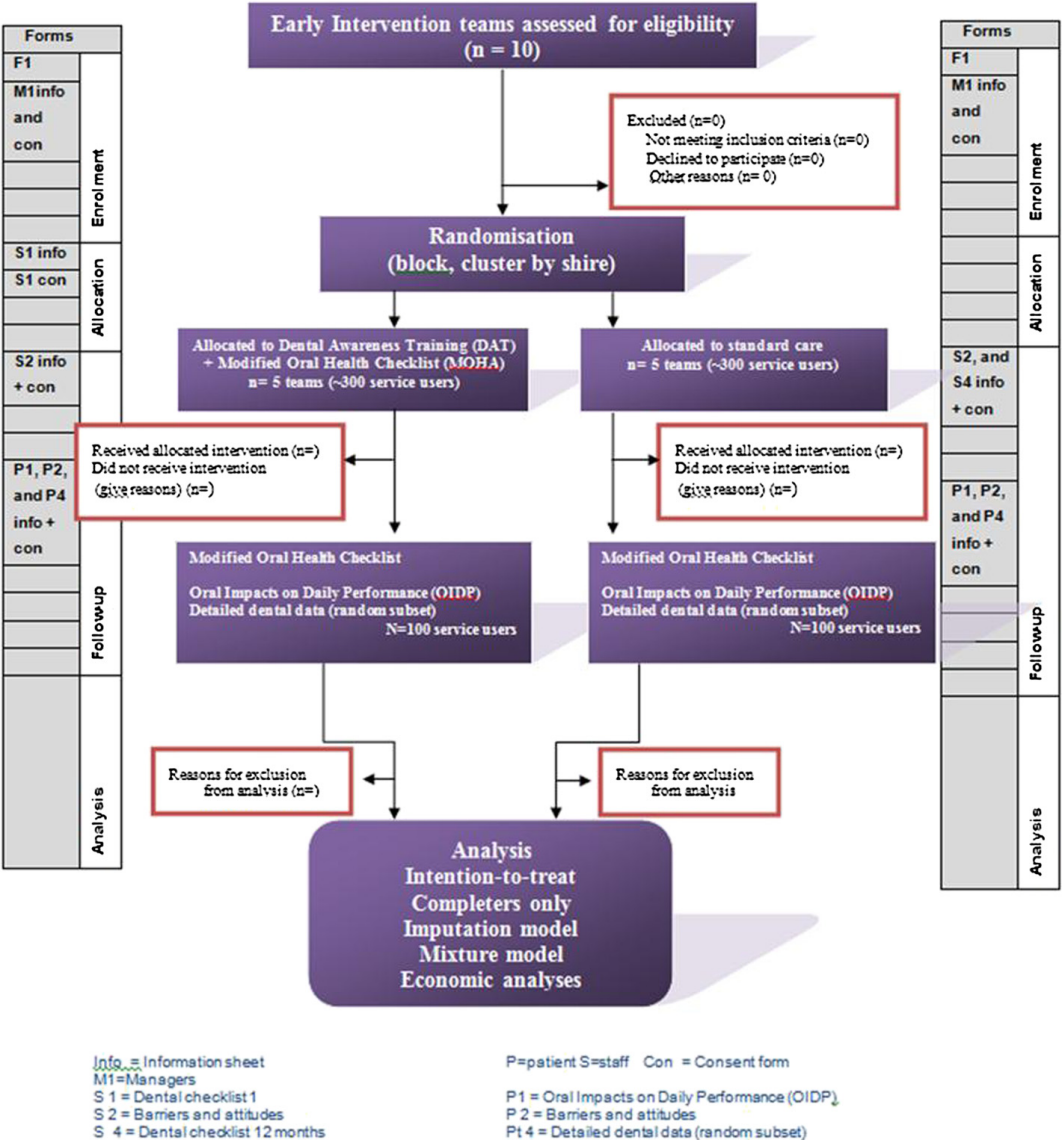
CONSORT flow diagram of participants through the trial.

All service users who have indicated that contact from the trial team is acceptable will be contacted by an independent researcher and consent gained for conduct. For the OIDP information sheets will be given out and consent forms will be signed. The OIDP will then be administered as a short 20-minute interview by the researcher. For the detailed dental data, information sheets will be given out and consent forms will be signed. Dentists will then be contacted and information about recent dental treatment and current state will be requested Figure 1.

### Serious unexpected events

Adverse events due to participating in this trial are not expected. Service users’ mental health will be closely monitored by their EIP teams, care co-ordinators will be asked to inform the trial team of any adverse event that either the care co-ordinator or service user feels was due to the checklist. The trial team will determine the seriousness and causality in conjunction with any treating medical practitioners. Serious adverse events are divided into two categories, those that seem likely to be independent of an oral checklist and those connected to oral healthcare. All adverse events will be recorded and closely monitored until resolution, stabilisation, or until it has been shown that involvement in the trial is not the cause by the data monitoring committee. All treatment-related serious adverse events will be recorded and reported to the REC as part of the annual reports. Unexpected serious adverse events will be reported to the REC by the chief investigator who will take appropriate medical action, which may include halting the trial and informing the sponsor of such action. If the event is determined to be due to involvement in the trial the REC will be informed using the reporting form found on the NRES web page within seven days of knowledge of the event. Within a further eight days, the trial team will send any follow-up information and reports to the REC. Any necessary amendments to the protocol will be made and the REC will be informed as required. Any participant who experiences an adverse event may be withdrawn from the study at the discretion of the investigator.

### Outcomes

#### Primary outcomes

Number of service users who have visited a dentist within 12 months of exposure to the checklist as reported on the checklist.

#### Secondary outcomes

Registered with a dentist, routine check-up within the last 12 months, owning a toothbrush, cleaning teeth twice a day, non-routine visit to a dentist in the last year, replacing existing toothbrush within the last six months, problems with mouth and teeth, OIDP. A cost analysis will also be carried out and other outcomes will include whether any service users have left the EIP service for any reason, including whether they refused to give consent, were discharged to another service, or whether they passed away.

### Data

#### Data analysis

This is a cluster randomised controlled trial with a small number of clusters but many participants within the clusters. We anticipate 600 to 800 dental checklists to be completed during the trial. The analysis will focus on whether the intervention has made a clinically significant difference to the data collected from the intervention teams (Additional file 5). The expected analysis will be a multilevel modelling or random effects methods. We will not analyse any difference between the care co-ordinators. . Data will be entered into a password-protected database with an audit trail.

#### Health economic analysis

This trial has been designed to be inexpensive to run but there will be some cost associated with training sessions and some staff time associated with completing the checklists, as well as addressing the dental needs of the service users. It is anticipated that more dental appointments will be made in the intervention teams, and the cost of these to the service user, EIP team and NHS dentist should be calculated.

There are two arms to this trial; the dental intervention delivered alongside standard care or standard care alone. The dental intervention includes a one-off session of dental awareness training provided to care co-ordinators within individual EIP teams, a dental information sheet provided to service users and a checklist that will be completed with each service user. This would thus lead to resource use including but not limited to planning, formulation, training and implementation of the dental awareness training as well as staff contact time for completion of checklists. Increase in level of staff input (contact time) as well as additional dental visits will need to be recorded. The cost-effectiveness analysis will be from the perspective of the National Health Service (NHS) in England, as we will plan on looking at the incremental cost-effectiveness ratio.

As this is the first pragmatic trial of oral health advice for people with serious mental illness, there is an uncertainty as to the magnitude of difference in effectiveness in the two arms of the trial. Should there be a statistically significant difference between the two groups, we would proceed with a full economic evaluation including cost-effectiveness analysis and cost-utility analysis (possible as the OIDP will be administered to a sample of 100 patients). However, should there be no difference, we would conduct a partial economic evaluation in the form of a cost analysis. The primary outcome measure for this trial is number of visits to the dentist within 12 months of exposure to the checklist.

Data regarding dental visits from a subsample of 100 service users will be obtained at the end of the study period and the difference in dental visits between the two groups can be used in calculation of the incremental cost-effectiveness ratio. The effectiveness data could also be calculated as a potential savings to the NHS for varied dental treatment due to early detection of dental pathology.

The estimated unit cost of the intervention and standard care will be based on 2012 to 2013 reimbursement rates for the NHS. The costs will be determined by multiplying the resources used and their volumes by the estimated cost per unit. A cost-effectiveness analysis is a form of economic evaluation that compares the relative costs and effects of two alternative options. In this trial the incremental cost-effectiveness ratio will be the ratio wherein the denominator would be patients keeping their dental appointments during the year (or Decayed, Missing and Filled Teeth (DMFT) rates if available) and the numerator would be the cost associated with the health gain. Based on data obtained, a detailed probabilistic sensitivity analysis will be carried out in order to get a range of incremental cost-effectiveness ratios.

#### Procedures for missing data

Some service users may leave the study early, but we plan to follow up as many as possible. We plan to carry out a sensitivity analysis and a selection model to test whether data missing is at random and whether the leaving early has an effect on treatment effects. To see if the data is missing at random, we will analyse whether it correlates to or is associated with the rest of the data. For data missing at random, a multiple imputation method will be used to fill missing values. If data missing is not at random or it may bias treatment effects, we shall run a selection-bias correction model (mixture model) to adjust for such bias.

#### Data monitoring

Monitoring of trial data includes confirmation of informed consent, source data verification, data storage and data transfer procedures, local quality control checks and procedures, back-up and disaster recovery of any local databases and validation of data manipulation. Entries on Case Report Forms (CRFs) will be verified by inspection against the source data. A sample of CRFs (10% or as per the study risk assessment) will be checked on a regular basis for verification of all entries made. In addition, the subsequent capture of the data on the trial database will be checked. Where corrections are required, these will carry a full audit trail and justification as to why amendments were made.

Trial data and evidence of monitoring and systems audits will be made available for inspection by the REC as required. In keeping with the GCP guidelines and in accordance with the University of Nottingham’s Research Code of Conduct, the chief investigator will maintain all records and documents regarding the conduct of the study, which will be kept for at least seven years or for longer if required. If the responsible investigator is no longer able to maintain the study records, a second person will be nominated to take over this responsibility. The trial master file and trial documents held by the chief investigator on behalf of the sponsor shall be finally archived at secure archive facilities at the University of Nottingham. This archive shall include all trial databases and associated meta-data encryption codes.

#### Governance

The Advisory Group is a multidisciplinary group to give advice and direction to the overall project and will meet every six weeks to begin with but will also provide continuing support and meet every few months for the duration of the trial. The Steering Group will meet twice a year to monitor the overall progress of the trial, adherence to the protocol and patient safety. The Data Monitoring Group will meet once a year to assess the progress and safety of the trial while providing the sponsor with recommendations regarding trial modification, continuation or termination. The Management Group consists of the trial team and will meet every week regarding the day-to-day running of the trial and data collection. This group will also communicate any unexpected events to both the Steering Group and the Advisory Group.

## Trial status

Recruitment started in February 2012. As of August 2012, 10 EIP teams caring for a total of 1,074 service users have been randomised. Recruitment for new trial sites is underway.

## Competing interests

The authors declare they have no competing interests.

## Authors’ contributions

HJ drafted the initial version of the protocol and coordinated revisions leading to the final manuscript for submission. CEA initiated the project. AC, JS and GT contributed to the methodology and supported the development of the project. PL and PC helped to initiate the project and supported the development of the project. MY and BG contributed to the statistical analysis section of the protocol and supported the development of the project. VF contributed to the health economic analysis section of the protocol and supported the development of the project. All authors read and approved the final manuscript.

## Supplementary Material

Additional file 1 Three shires dental checklist. Click here for file

Additional file 2 **Three shires early intervention dental trial.** Dental awareness training manual. Click here for file

Additional file 3 Promoting healthy teeth and gums. Click here for file

Additional file 4 Oral Impacts on Daily Performance (OIDP): interviewer-administered questionnaire. Click here for file

Additional file 5 **Dummy tables.** Primary outcomes. Secondary outcomes. Click here for file
