# Supplementary material for: An oral health intervention for people with serious mental illness (Three Shires Early Intervention Dental Trial): study protocol for a randomised controlled trial
Source: Trials. 2013 May 29;14:158. doi: 10.1186/1745-6215-14-158 (PMC3669616; doi:10.1186/1745-6215-14-158)
Supplement: Additional file 1 — Three shires dental checklist. [file 1745-6215-14-158-S1.pdf]

Three Shires Dental Checklist

THE THREE SHIRES • EARLY • INTERVENTION DENTAL TRIAL

ID

Date _ _ / _ _ / 20 _ _

History

Age: _ _ Years

Sex: Male ☐ Female ☐

Considering your total clinical experience with this particular population, how mentally ill is this person at this time?

1=not at all ill ☐ 2=borderline mentally ill ☐ 3=mildly ill ☐ 4=moderately ill ☐

5=markedly ill ☐ 6=severely ill ☐ 7=among the most extremely ill ☐

Dentist

Is the client registered with a dentist? Yes ☐ No ☐ Do not know ☐

When did the client last see a dentist? (nearest month and year)_ _ /_ _Do not know ☐

Was this visit: a routine check-up ☐ to fix a problem ☐ both ☐ Do not know ☐

If the person has not seen a dentist, what stopped them?

Toothbrush

Does the client have a toothbrush? Yes ☐ No ☐ Do not know ☐

Lots of people have a brush and do not use it, how often do they brush their teeth?

When did they last change it for a new one?

Current state

How many adult teeth has the client had removed because they were bad?

In the past 6 months, has the client had any difficulty due to problems with their mouth and teeth (or dentures)? Yes ☐ No ☐ Do not know ☐

If 'YES' to last question - what was/is the main difficulty? (suggestions overleaf)

Again, if 'YES' to last question - what problems did this cause? (suggestions overleaf)

Does the client need urgent dental treatment? Yes ☐ No ☐ Do not know ☐

Thank you for filling in this form
- please file one copy with CPA documents
- please post one copy to the trial team in the envelope provided
- please give the client the Information Leaflet if they want one

CLAHRC Nottinghamshire, Derbyshire, Lincolnshire
the institute of mental health
The University of Nottingham UNITED KINGDOM · CHINA · MALAYSIA

Suggestions for main difficulty

bad breath
bleeding gums
clicking or grating noise in jaw
colour of teeth
deformity of mouth or face (e.g. cleft lip, cleft palate)
fractured tooth
improper filling or crown (e.g. broken, colour)
loose or ill-fitting denture
loose tooth
oral ulcer or spot
orthodontic appliance
position of teeth (e.g. crooked or projecting, gap)
receding gums
sensitive tooth
shape or size of teeth
swollen gums (gum abscess)
tartar
tooth decay (hole in tooth)
tooth loss
toothache

Suggestions for problems that the oral health difficulty may cause

eating food
speaking clearly
cleaning your teeth (dentures)
doing light physical activities, such as housework
going out, for example to shop or visit someone
sleeping
relaxing
smiling, laughing and showing teeth without embarrassment
with your emotional state, for example becoming more easily upset than usual
carrying out your major work
enjoying the contact of other people, such as relatives, friends or neighbours
