# Supplementary material for: An oral health intervention for people with serious mental illness (Three Shires Early Intervention Dental Trial): study protocol for a randomised controlled trial
Source: Trials. 2013 May 29;14:158. doi: 10.1186/1745-6215-14-158 (PMC3669616; doi:10.1186/1745-6215-14-158)
Supplement: Additional file 2 — Three shires early intervention dental trial. Dental awareness training manual. [file 1745-6215-14-158-S2.docx]

**
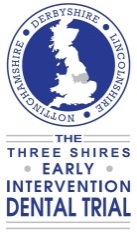
Three Shires Early Intervention Dental Trial**

**Dental Awareness Training Manual**

Topics to cover:

- The trial was set up in 2009 with funding by the National Institute for Health Research through the Collaboration for Leadership in Applied Health Research and Care for Nottinghamshire, Derbyshire and Lincolnshire.
- The aim of the trial is to see whether a simple checklist can improve the oral health of people with serious mental illness.
- The oral health of people with serious mental illness has generally been found to be worse than that of the general population due to side effects of medication, issues with self-care, barriers to treatment and poor recognition of dental problems.
- Guidelines suggest oral health should be monitored for people with mental illness but this is often not reflected in current practice and a Cochrane systematic review revealed no existing randomised trials of oral health advice for people with serious mental illness.
- Early Intervention in Psychosis (EIP) teams are randomly allocated to receive the dental intervention or continue with standard care for 12 months. All service users cared for by the team aged >18 years old are eligible.
- The dental checklist was adapted from existing guidelines in collaboration with clinicians and service users and comprises questions regarding service users’ current oral health state and practice, and general mental state.
- Care Co-ordinators (CCO) answer the history question themselves and then ask service users the remaining questions concerning professional dental care, oral hygiene practice and current oral state.
- If a service user is not happy to take part, the CCO writes the ID number on the checklist and posts the blank form back to the trial team in the prepaid envelopes provided.
- ID numbers are made up of a letter to identify the team, then the CCO initials then 3 unique numbers for service user – e.g ABC123. An electronic spreadsheet containing ID numbers for each CCO will be emailed to the team after the training session so that a record of which ID number has been assigned to which service user can be held by the team. No person identifiable information is to be sent to the trial team.
- The CCOs keep the colourful top sheet from the checklist in the service users’ notes and post the trial team the yellow carbon copy in the prepaid envelopes provided.
- The CCOs provide a copy of the information sheet to service users if they would like one as this includes oral hygiene advice and information regarding finding an NHS dentist.
- If any adverse events occur that are thought to be due to the dental checklist, CCOs should notify the trial team via the contact details provided on the information sheets.
- CCOs will be asked to read the information sheet about the study and sign the consent form.
