# Supplementary material for: An oral health intervention for people with serious mental illness (Three Shires Early Intervention Dental Trial): study protocol for a randomised controlled trial
Source: Trials. 2013 May 29;14:158. doi: 10.1186/1745-6215-14-158 (PMC3669616; doi:10.1186/1745-6215-14-158)
Supplement: Additional file 3 — Promoting healthy teeth and gums. [file 1745-6215-14-158-S3.doc]

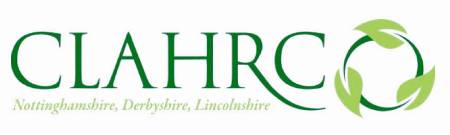


Promoting healthy teeth and gums

**Brushing your teeth is a good idea**

- **Brush your teeth twice a day with a fluoride toothpaste**
- **After cleaning teeth spit out – BUT do not rinse with water**

**Drugs (prescribed and illegal) can affect your teeth**

- **For drugs given as syrups - always ask for sugar-free or rinse mouth with water**
- **If you suffer from dry mouth - try sugar-free chewing gum**


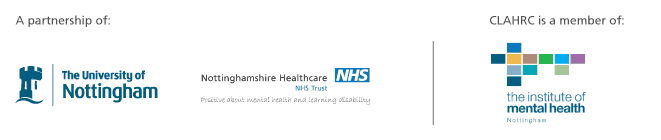


**Diet is important**

- **Try and eat a healthy balanced diet (veg, fruit – that sort of thing)**
- **Limit sugary food and drinks to mealtimes only**

**Other stuff**

**Smoking and alcohol increase the chance of mouth cancer.**

**Bleeding gums are not good**

- **If bleeding continues ask a dentist for advice**
