# Supplementary material for: An oral health intervention for people with serious mental illness (Three Shires Early Intervention Dental Trial): study protocol for a randomised controlled trial
Source: Trials. 2013 May 29;14:158. doi: 10.1186/1745-6215-14-158 (PMC3669616; doi:10.1186/1745-6215-14-158)
Supplement: Additional file 4 — Oral Impacts on Daily Performance (OIDP): interviewer-administered questionnaire. [file 1745-6215-14-158-S4.pdf]

ORAL IMPACTS ON DAILY PERFORMANCE (OIDP): INTERVIEWER-ADMINISTERED QUESTIONNAIRE

THE THREE SHIRES EARLY INTERVENTION DENTAL TRIAL

ID

This card shows some everyday activities / behaviours. I would like you to tell me whether or not problems with your mouth and teeth (or dentures) have caused you difficulty with each one of those in the past 6 months.

YES	NO	Regular basis	Part of period	How often?	How much?
Eating food	1	2	1 → Q3	2 → Q4	
Speaking clearly	1	2	1 → Q3	2 → Q4	
Cleaning your teeth (dentures)	1	2	1 → Q3	2 → Q4	
Doing light physical activities, such as housework	1	2	1 → Q3	2 → Q4	
Going out, for example to shop or visit someone	1	2	1 → Q3	2 → Q4	
Sleeping	1	2	1 → Q3	2 → Q4	
Relaxing	1	2	1 → Q3	2 → Q4	
Smiling, laughing and showing teeth without embarrassment	1	2	1 → Q3	2 → Q4	
With your emotional state, for example becoming more easily upset than usual	1	2	1 → Q3	2 → Q4	
Carrying out your major work	1	2	1 → Q3	2 → Q4	
Enjoying the contact of other people, such as relatives, friends or neighbours	1	2	1 → Q3	2 → Q4	

Q1. In the past 6 months, have you had any difficulty ... ACTIVITY / BEHAVIOR... due to problems with your mouth and teeth (or dentures)?
CODE "YES" OR "NO". FOR EACH ACTIVITY / BEHAVIOR CODED `YES', ASK Q2-Q6.

Q2. Have you had this difficulty ... ACTIVITY / BEHAVIOR... on a regular basis over the past 6 months or only for part of this period?

on a regular basis	1 **ASK Q3**
only for part of this period	2 **GO TO Q4**

CODE ONE ONLY, THEN ASK Q3 OR Q4 AS INDICATED.

IF RESTRICTED "ON A REGULAR BASIS" (CODE 1 AT Q2)
Q3. During the past 6 months, how often have you had this difficulty .ACTIVITY / BEHAVIOR...?

every day or nearly every day	5
about 3-4 times a week	4
about 1-2 times a week	3
about 1-2 times a month	2
or less often than once a month	1
(Can't say)	9

ENTER ANSWER CODE IN BOX UNDER Q3 ON GRID. GO TO Q5.

ORAL IMPACTS ON DAILY PERFORMANCE (OIDP): INTERVIEWER-ADMINISTERED QUESTIONNAIRE

ID

IF RESTRICTED "ONLY FOR PART OF THIS PERIOD" (CODE 2 AT Q2).

Q4. For how much of the past 6 months have you had this difficulty...ACTIVITY / BEHAVIOUR..?

for more than 3 months	5
for more than 2, up to 3 months	4
for more than 1, up to 2 months	3
for more than 5 days, up to a month	2
or for 5 days or less?	1
(Can't say)	9

Q5. And using a scale from 0 to 5, where 0 is no effect and 5 a very severe effect, how much effect would you say

Q6. Now, I am going to investigate the specific condition that caused this difficulty. Which one of the following oral conditions has caused this difficulty ...ACTIVITY / BEHAVIOR...?

toothache	1	receding gums	11
sensitive tooth	2	tartar	12
tooth decay (hole in tooth)	3	oral ulcer or spot	13
fractured tooth	4	bad breath	14
tooth loss	5	deformity of mouth or face (e.g. cleft lip, cleft palate)	15
loose tooth	6	clicking or grating noise in jaw	16
colour of teeth	7	improper filling or crown (e.g. broken, colour)	17
position of teeth (e.g. crooked or projecting, gap)	8	loose or ill-fitting denture	18
shape or size of teeth	9	orthodontic appliance	19
bleeding gums	10	or any other reason? (please specify)	88
