# Supplementary material for: An oral health intervention for people with serious mental illness (Three Shires Early Intervention Dental Trial): study protocol for a randomised controlled trial
Source: Trials. 2013 May 29;14:158. doi: 10.1186/1745-6215-14-158 (PMC3669616; doi:10.1186/1745-6215-14-158)
Supplement: Additional file 5 — Dummy tables. Primary outcomes. Secondary outcomes. [file 1745-6215-14-158-S5.docx]

Additional file 2

**Dummy Tables**

**Primary Outcomes**

|  | **Dental awareness training + dental checklist**  **(n = …)** | | | | **Standard care**  **(n = …)** | | | |
| --- | --- | --- | --- | --- | --- | --- | --- | --- |
|  | ITT | Completers only | Imputation model | Mixture model | ITT | Completers only | Imputation model | Mixture model |
| **Visited dentist within last 12 months (%)** |  |  |  |  |  |  |  |  |

**Secondary outcomes**

|  | **Dental awareness training + dental checklist**  **(n = …)** | | | | **Standard care**  **(n = …)** | | | |
| --- | --- | --- | --- | --- | --- | --- | --- | --- |
|  | ITT | Completers only | Imputation model | Mixture model | ITT | Completers only | Imputation model | Mixture model |
| **Registered with dentist (%)** |  |  |  |  |  |  |  |  |
| **Routine check-up within last 12 months (%)** |  |  |  |  |  |  |  |  |
| **Owning a toothbrush (%)** |  |  |  |  |  |  |  |  |
| **Cleaning teeth twice a day (%)** |  |  |  |  |  |  |  |  |
| **Lost to follow up (%)** |  |  |  |  |  |  |  |  |
| **Refusing to participate in follow up (%)** |  |  |  |  |  |  |  |  |
| **Refusing OIDP follow up (%)** |  |  |  |  |  |  |  |  |
| **Non-routine visit to a dentist in last year (%)** |  |  |  |  |  |  |  |  |
| **Replacing existing toothbrush within the last six months (%)** |  |  |  |  |  |  |  |  |
| **Problems with mouth and teeth (%)** |  |  |  |  |  |  |  |  |
| **Oral Impacts on Daily Performance (OIDP)**  **- overall score (mean, SD)** |  |  |  |  |  |  |  |  |
| **At least fairly severe functional difficulty on regular basis (%)**  e.g. Eating food  Speaking clearly  Cleaning your teeth (dentures)  Doing light physical activities, such as housework  Going out, for example to shop or visit someone  Sleeping  Relaxing  Smiling, laughing and showing teeth without embarrassment  With your emotional state, for example becoming more easily upset than usual  Carrying out your major work  Enjoying the contact of other people, such as relatives, friends or neighbours |  |  |  |  |  |  |  |  |
|  |  |  |  |  |  |  |  |  |
